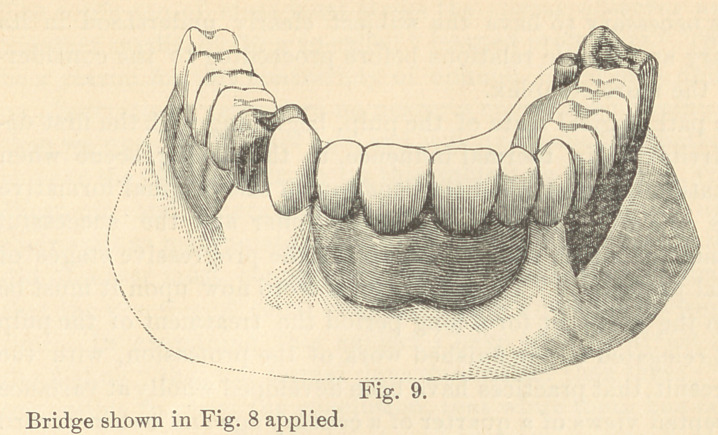# Removable Bridgework

**Published:** 1889-04

**Authors:** T. S. Waters

**Affiliations:** Baltimore


### REMOVABLE BRIDGEWORK[1].

BY. T. S. WATERS, D.D.S., BALTIMORE.

In addressing you on this occasion I feel that I am speaking on a subject that while really old, is yet comparatively new.

Bridgework was made many years ago, but it was not then known by that name. Our professional predecessors made bridgework decades ago by inserting gold plates with teeth on them and retaining them in the mouth by two or more gold pins soldered to the plate and extending into the roots left in the mouth. This was primitive bridgework, but nevertheless it *was* bridgework. Therefore the subject is an old one.

But improvements have been made in the methods of applying bridgework, which have changed its character and features,—without changing its basal principle,—and to that exent it is new.

The bridgework of the present day may be divided into two classes : the rigid or permanent, which cannot be removed without a special operation by the dentist ; and the removable, which can be removed and replaced at the pleasure of the wearer.

The ancient bridgework before spoken of, belongs to the former class, but of late years bands, ferrules or caps enclosing the roots at the neck, or the remaining natural crowns have been substituted for the pins extending into the roots and this constitutes one of the new features of bridgework. Another new feature of bridgework, when rigid or permanent, is the dispensing with the plate formerly used. This was considered necessary from a cleanly and therefore hygienic point of view, because a plate covering a considerable portion of mucous membrane and not removable, would, of course, allow the collection and retention of food in a semi-fluid state between the two surfaces, which would not only be uncleanly, but would by its constant presence affect injuriously the sanitary condition of the mucous membrane with which it is in contact. To further avoid the bad results of having any portion of the mucous membrane covered permanently with a plate, the device was adopted of cutting away the lingual or palatine portion of the artificial teeth next the gum, so that only the base of the

[1] Read at the tenth aniversary meeting of the Odontological Society of Pennsylvania, Dec. 13, 1888.

labial or buccal sides should come in contact with the gum. This, it was thought, would not only secure the healthy condition of the mucous membrane, but would also make the piece self-cleansing; but it is doubtful if that object is accomplished, since the surfaces which are inaccessible to the mechanical action of the food in mastication or of the tooth brush in cleaning, will in many mouths, be found always covered with a deposit, nor can the requirement of thorough cleanliness be fully met in any case in which the piece cannot be removed and properly cleaned. I beg to be understood here as referring to bridgework only, and not to single crown cases, because in the latter every part is accessible to the mechanical action of the food and the brush, as are the natural teeth.

As we have said that in permanent bridgework the plate is dispensed with for purposes of cleanliness and hygiene, it is proper here to discuss, to some extent, the functions that a plate performs as a bearer of artificial teeth.

It is sufficient for present purposes to speak of only one of these functions. It is the experience of all dentists that when a plate is used with a small surface, the pressure that is brought to bear upon it in the process of mastication, causes a rapid absorption of the subjacent tissues both soft and hard and it has been found necessary in the insertion of a small number of teeth, in order to avoid as far as possible this bad result, to make the plate as broad as the case will admit, thus distributing, what may in such cases be called a fixed amount of pressure for each case, over a broader mucous surface lessening the evil from absorption; and I would suggest that in all cases where plates are used this pressure should, for obvious reasons, be so distributed as to bring as great a proportion of it over the hard or true bone, and as small a proportion over the softer alveolar structure as the case will admit.

The necessity of guarding this point will be better appreciated if we give due consideration to the very great force exerted by the masseter muscles in mastication.

One of the offices, then, and a very important one, of a plate bearing artificial teeth is to so apply, divide and distribute the pressure on the mucous surface as to produce the least possible injury to the subjacent tissues, and I consider this point of so great importance that I hold that it is wrong to dispense with the plate (as is done in permanent bridgework) for the reason that the use of a plate in bridgework relieves the teeth and roots to which the denture is attached of much of the strain that is brought to bear in mastication, and transfers and distributes it over the mucous surface.

Dr. E. C. Kirk, in his paper on the "Hygienic relations of artificial dentures," page 1022, vol. 2, of American System of Dentistry, sets forth plainly and, in my opinion, correctly the objections to a certain class of bridgework, which can only be said of that which is permanent and without plates. But the bad results spoken of in these objection are, as I believe, fully met and avoided in the system that I have the honor and pleasure now to present to you, viz.: that of removable bridgework, because in it the denture is retained securely and steadily in the mouth yet is readily removed and replaced at pleasure by the wearer,—the pressure and strain are distributed properly over all structures and tissues available for that purpose, and the roots and crowns to which the denture is attached are so prepared that there is no place for the lodgement and retention of food, and when the denture is removed, both it and the mouth can be thoroughly cleansed.

It is also so evident as not to require explanation that should the roots or other tissues be attacked by disease, thus requiring treatment, or should repairs to the mechanism become necessary, the removable bridgework offers facilities for those purposes not to be found in that which belongs to the permanent class.

I do not claim, that the idea of removable bridgework is original with me, but I do assert and claim that I constructed the first really practical piece of bridgework of which the wearer had perfect control as to removal and replacement, and in which, by the means adopted, the best possible hygienic condition is attained.

Having accomplished this much I was led to devote my studies and energies to the combination and application of such devices as would best effect these desirable results. Most of these devices were known to the profession, but their combination and application as now presented to you, I believe to be new in bridgework, and to these devices and to dentures illustrating their character and application, I call your attention.

These devices are three in number, each one of which may be used alone or two or all three may be combined in the same case, and applied as the position, character and relation of the teeth and roots remaining in the mouth may seem to indicate.

The first one that I bring to your notice is a gold crown fitted to and sliding on a cap attached permanently to the root or natural crown. This cap is made high and has on one side a longitudinal groove. See Fig. 1. The gold crown has soldered on the inside a spring catch, which works in the groove on the cap and holds the crown firmly in its place. See Fig. 2.

Fig. 1.

Cast of practical case showing bicuspid and molar, capped ready for the application of bridge. The longitudinal slot or groove is seen on the mesial face of the bicuspid.

Fig. 2.

Bridge-piece ready to apply. The outlined figure represents the capped teeth shown in cut No. 1.

This device may be used (though I do not recommend it) in simple crown cases, and allows the removal of the crown at pleasure for the purpose of cleaning. It will be readily seen that, under proper circumstances, two or more roots or teeth, may be fitted with this device, the gold crowns being soldered to and made a part of the denture, making the whole a piece of bridgework capable of being removed, cleaned and replaced at the pleasure of the wearer. the spring catch regulates the firmness of retention. See Fig. 3.

Fig. 3.

The above piece of bridge-work applied.

This is my own invention, and I obtained letters patent for it in order that I might secure it and donate it to the profession, which I have done.

Fig. 4.

Cast of case showing six anterior teeth prepared for the application of the split-post attachment.

The next device is the box cap and split post, the box cap being fitted permanently to the root and the split post being soldered to the plate bearing the teeth. The box cap is the usual cap with a box or tube soldered to it and extending into the root, the cap end of the tube being open. The split post is so secured to the denture

Fig. 5.

Piece of bridge-work with split pins ready for application.

as to slide snugly into this tube, the firmness of retention being regulated by pressing the split slightly open when necessary. This device like the first may, under proper circumstances, be used altogether in any one case as shown in Figs. in which case the whole denture is supported by box caps and split posts adjusted to the roots of the six anterior teeth.

Fig. 6.

The above piece of work applied.

The third device is that of soldering to the side of the gold crown covering the natural tooth, a split pin or post, which is inserted into the open tube attached to the denture. See molar tooth Fig. 7. This device, like the others, can be used alone in any one case.

This device, like the others, can be used alone in any one case

Fig. 7.

Case where both the preceding features are used. In this case, however, the split pin is soldered to the tooth cap.

as illustrated in Dr. George Evan's "Practical Treatise on Artificial Crown and Bridgework," page 184, figures 383, but which is credited to Dr. H. A. Parr.

As before remarked these devices may be used singly or in

Fi.g 8.

Piece ready for application.

combination in any one case. In one of the dentures, Fig. 6, submitted to you the box, cap and split post alone are used; in another, Fig. 1., the cap, gold crown and spring catch only are used in another, Fig. 7, the three are applied, in which the entire denture is attached to and retained by two cuspids, a bicuspid and a molar.

Fig. 9.

Bridge shown in Fig. 8 applied.

In all this, great care must, of course, be taken, in the preparation of the roots and natural crowns to protect them against the action of destructive agents.

With this preparation of the roots and natural teeth with the proper adaptation of these devices, and with the use of as large a surface of plate as the case will admit, your speaker is at a loss to conceive why removable bridge work should not become the work of the future, and he leaves the subject for your consideration with the remark, that the use of removable bridgework, of the character and with the features above presented, will relieve the dental profession from the domination of the International Tooth Crown Co., which it is trying to exercise by virtue of its ownership of the "Lowe Patent."

## Figures and Tables

**Fig. 1. f1:**
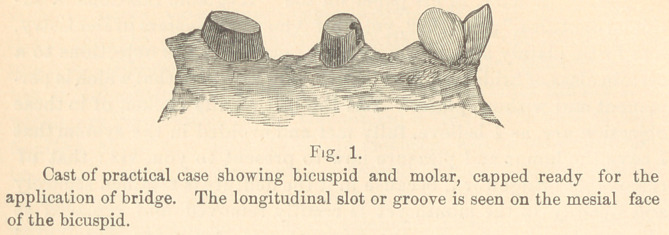


**Fig. 2. f2:**
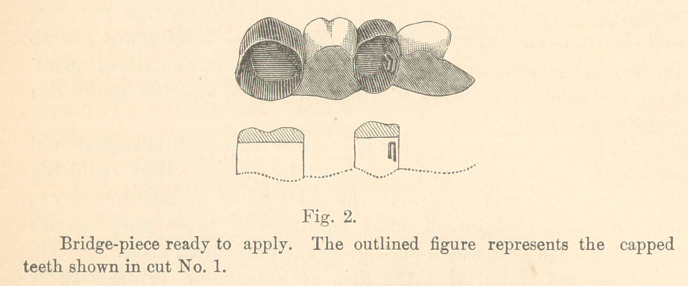


**Fig. 3. f3:**
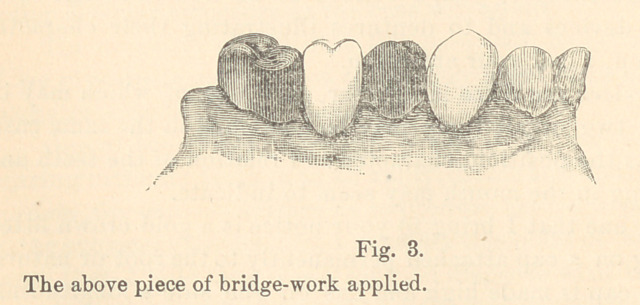


**Fig. 4. f4:**
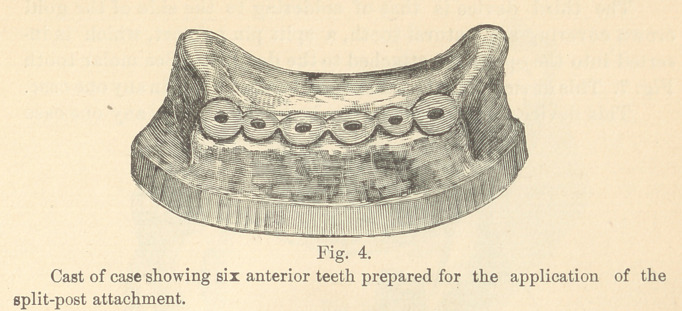


**Fig. 5. f5:**
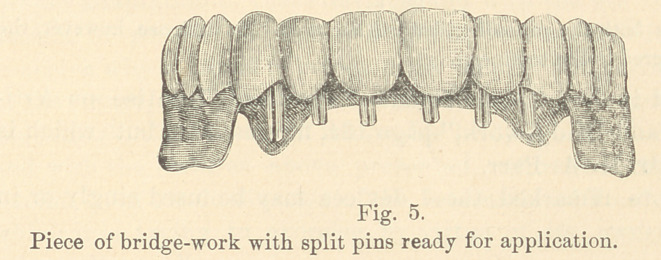


**Fig. 6. f6:**
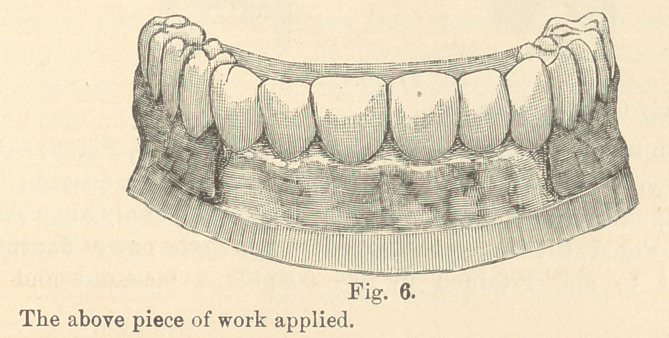


**Fig. 7. f7:**
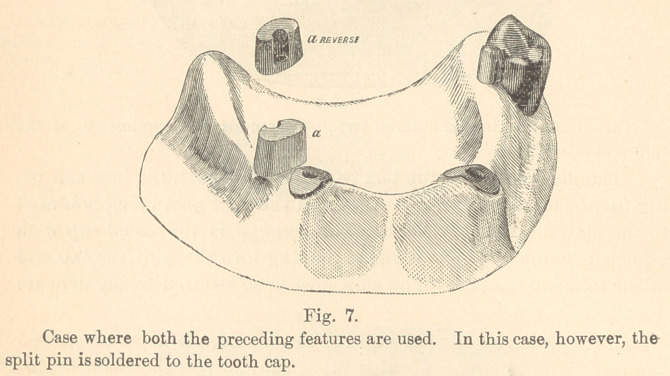


**Fig. 8. f8:**
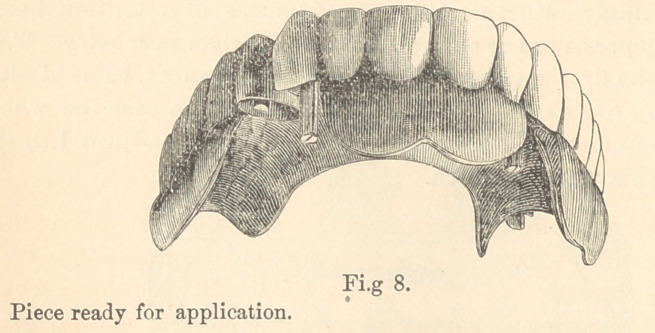


**Fig. 9. f9:**